# Can a single interactive seminar durably improve knowledge and confidence of hospital diabetes management?

**DOI:** 10.1186/s40842-016-0038-4

**Published:** 2016-12-01

**Authors:** Timothy W. Bodnar, Jennifer J. Iyengar, Preethi V. Patil, Roma Y. Gianchandani

**Affiliations:** 1 24 Frank Lloyd Wright Drive, P.O. Box 482, Ann Arbor, MI 48106 USA; 2 Ann Arbor Endocrinology & Diabetes Associates P.C., Ypsilanti, MI USA; 3 University of Michigan Health System, Ann Arbor, MI USA

**Keywords:** Diabetes, Glycemic Management, Medical Education, Medical Students

## Abstract

**Background:**

Safe and effective diabetes management in the hospital is challenging. Inadequate knowledge has been identified by trainees as a key barrier. In this study we assess both the short-term and long-term impact of an interactive seminar on medical student knowledge and comfort with hospital diabetes management.

**Methods:**

An interactive seminar covering hospital diabetes management and utilizing an audience response system was added to the third-year medical student curriculum. Students were given a multiple choice assessment immediately before and after the seminar to assess their comprehension of the material. Students were also asked to rate their confidence on this topic. Approximately 6 months later, students were given the same assessment to determine if the improvements in hospital diabetes knowledge and confidence were durable over time. Students from the preceding medical school class, who did not have a hospital diabetes seminar as a part of their curriculum, were used as a control.

**Results:**

Fifty–three students participated in the short-term assessment immediately before and after the seminar. The mean score (maximum 15) was 7.7 +/- 2.7 (51%) on the pre-test and 11.4 +/- 1.8 (76%) on the post-test (*p* < 0.01). 75 students who attended the seminar completed the same set of questions 6 months later with mean score of 9.2 ± 2.3 (61%). The control group of 100 students who did not attend seminar had a mean score of 8.8 ± 2.5 (58%). The difference in scores between the students 6-months after the seminar and the control group was not significantly different (*p* = 0.30).

**Conclusions:**

Despite initial short-term gains, a single seminar on hospital diabetes management did not durably improve trainee knowledge or confidence. Addition of repeated and focused interactions during clinical rotations or other sustained methods of exposure need to be evaluated.

**Electronic supplementary material:**

The online version of this article (doi:10.1186/s40842-016-0038-4) contains supplementary material, which is available to authorized users.

## Background

Patients with diabetes account for a disproportionally high percentage of inpatient stays, estimated at 22% of hospital inpatient days in the United States [1]. Both hyperglycemia and hypoglycemia remain common problems among admitted patients with diabetes and have been associated with poor clinical outcomes [2, 3]. Therefore, effective glycemic management in the hospital is an important safety and quality care measure for patient outcomes. The management of diabetes in the inpatient setting poses several unique challenges including fluctuating nutritional status, confounding medications, and presence of other acute and chronic illnesses. Insulin is one of the most common drugs implicated in preventable adverse drug events in the hospital [4]. As the prevalence of diabetes continues to grow, it is imperative that trainees, regardless of fields of practice, are well versed in diabetes management.

Although resident physicians acknowledge the importance of glycemic management in the hospital, there are several identifiable barriers to the attainment of target glucose levels [5–7]. One of the cited barriers to improved inpatient glycemic management among residents is lack of knowledge of appropriate insulin regimens [6, 7]. A survey of medicine residents found that less than half reported that hospital diabetes management was explicitly addressed in their residency and 97% of responded that they would like this training to be included in the curriculum [8]. This finding is not unique to a single instituition or even the United States, with several studies nationally and internationally noting concerns about the comfort and preparedness of physicians and trainees to manage inpatient diabetes [9–11].

Several previously published interventions have targeted trainees at resident physician level in an effort to improve trainee knowledge of inpatient glycemic management. Such interventions have incorporated a variety of educational formats including the use of computer-based modules [12, 13], case-based training [14], mobile device-based educational tool [15], and a comprehensive longitudinal curriculum [16]. While many of these studies had positive outcomes as measured by improvement in diabetes knowledge and comfort among trainees or improvements in measured glycemic control on the wards, there is a limited data on the long-term durability of any improvements. Similarly, short-term medical education interventions have been studied for non-diabetes topics, but have also failed to examine long-term retention [17–19]. Many of the previous diabetes studies focused only on internal medicine resident physicians, despite evidence that trainees in other specialities demonstrate less knowledge of hospital diabetes management [10]. Given the rise of diabetes and hyperglycemia in the hospital, it is increasingly important that trainees of many specialties become knowledgeable and comfortable with hospital diabetes management.

We hypothesized that targeting students earlier in their medical training would help reduce the gap in hospital diabetes knowledge across specialties. However, in order for such an intervention to be effective beyond medical school, students would need gains in knowledge and confidence to be durable. Similar to studies of trainees at the resident physician level, there is evidence that medical students also lack knowledge in hospital diabetes. A study by Landsang et al of fourth year medical students found notable knowledge gaps including failure to recognize stress hyperglycemia and frequent recommendation the use of sliding scale insulin without scheduled basal bolus insulin [20]. This study also found students were less likely to provide appropriate management of diabetes than they were to other commonly encountered clinical problems such as chest pain or hypertension. A well-designed study by MacEwen et al found that a “Diabetes Day” with lectures and learning tutorials improved diabetes knowledge and comfort in medical students in the UK [21]. However, they did not look at long-term maintenance of knowledge. We developed an interactive seminar on hospital diabetes management for the third-year medical student curriculum and evaluated if this would durably improve knowledge and confidence related to this topic.

## Methods

### Setting and population

The University of Michigan Medical School (UMMS), in Ann Arbor, MI, USA, enrolls approximately 170–175 students per class per year, for a combined enrollment between 650 and 710 students during a particular academic year. In-state students vary between approximately 45–55% for a given class, and the percentage of female students has ranged from 45 to 55% over fiscal years 2011–2015 [22]. UMMS students match into a wide variety of postgraduate training programs, with the top 5 (in descending order) from 2008-2012 being: Internal Medicine, Pediatrics, Emergency Medicine, Anesthesiology, and Family Practice [23].

Through at least the 2013–2014 academic year, the curriculum at UMMS consists of 2 years of pre-clinical training (in classroom and laboratory settings) and 2 years of clinical training (in patient care settings) including required clerkships such as Surgery, Obstetrics & Gynecology, Psychiatry, and Internal Medicine. During the entirety of the M3 year, M3 students have protected time on Friday afternoons for a mandatory lecture series entitled, “The M3 Seminar Series.” All M3 students, regardless of current clerkship or rotation, meet for a series of seminars on important medical and humanistic topics. The curriculum for the M3 Seminar Series has evolved over time in response to changing educational needs, student evaluations of individual seminars, and student and faculty requests for individual topics. The first seminar given on hospital diabetes management was given during the 2012–2013 academic year, meaning that the M3 students graduating in 2014 attended this seminar, while the M4 students graduating in 2013 (who were M3 students during the 2011–2012 academic year) did not experience this seminar or anything similar.

The M3 seminar covering hospital diabetes management is the only formal didactic experience on this topic that all UMMS students receiving during their clinical training (M3 and M4 years). All other training on this topic is less formal and more experiential (“on the job” training during clinical clerkships, subinternships, and electives), and thus, may be more variable from student to student.

### Intervention

**Fig. 1:**
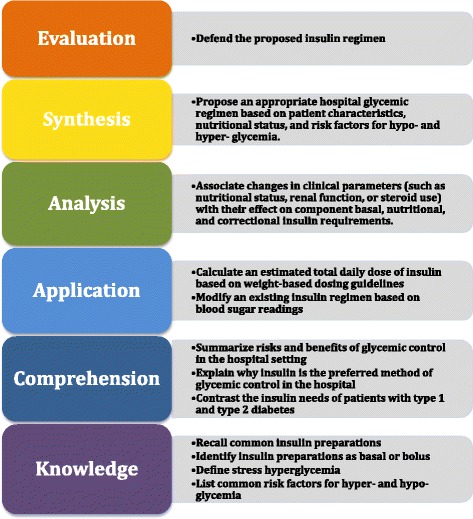
Educational objectives for the seminar organized by Bloom’s Taxonomy

The authors created an approximately 90-min interactive session, utilizing didactic slides covering important concepts necessary for safe and effective hospital diabetes management (including an evidence-based approach) of non-critically ill patients, as well as a series of interactive cases to illustrate some key concepts. The interactive cases incorporated an electronic audience remote response system to encourage audience participation. This presentation underwent a series of edits amongst the authorship group, with input from colleagues in the Division of Metabolism, Endocrinology, and Diabetes within the Department of Internal Medicine at UMMS. This seminar had a didactic component taught by an endocrinology faculty member (RYG) and the cases by endocrinology trainees (TWB and JJI). This presentation has been published online [24]. The educational objectives of the seminar are listed in Fig. 1 using Bloom’s Taxonomy.

### Assessment

The assessment tool was also developed by the authors. Although several excellent assessment tools have been published in the past by other groups [13, 16, 21, 25, 26], the authors felt it was important to tailor the content of the assessment to the material in the presentation, which covered non-acute inpatient diabetes management and did not include acute topics like DKA or HHS or more advanced resident-level topics like peripartum glycemic management. The 15 multiple-choice questions (each with one correct answer choice and 3 incorrect answer choices) were formulated to cover critical pieces of knowledge as deemed important by the authors and colleagues, with combined decades of experience managing diabetes in the hospital setting. The 3 questions asking participants to rate their confidence managing diabetes, blood pressure, and electrolyte disturbances in the hospital were chosen to assess whether the addition of the seminar improved confidence managing diabetes (experimental group compared to control group) and whether any improvement was seen relative to other problems often managed in the hospital setting (but which, like type 2 diabetes, are much less often the primary reason for admission).

Questions were vetted through the assistance of the Medical Education Scholars Program, a faculty development seminar for expertise in medical education at UMMS. Writing quiz/survey questions is a key part of the curriculum for this group. Questions deemed confusing or vague by the group (consisting of UMMS faculty members in a variety of medical specialties) were re-written or discarded. Finally, colleagues in the Division of Metabolism, Endocrinology, and Diabetes within the Department of Internal Medicine at UMMS also reviewed the questions. See Additional file 1 for multiple choice questions, answer choices, and correct answers.

### Experimental group

The experimental group consisted of students at UMMS during the 2013–2014 academic year (2013–2014 cohort) who attended the M3 seminar covering hospital diabetes management. Short-term changes in knowledge among the experimental group were assessed by adminstering the assessment tool immediately before and after the seminar, with responses collected using an audience reponse system. Although students were required to attend the seminar as part of their M3 curriculum, partipation in the pre- and post- assessment was voluntary. In order to assess the long-term impact of the seminar, students who attended the M3 seminar were recruited via group email approximately 6-months after seminar completion for reassessment. Any student who attended the seminar was allow to complete the long-term assessment, not just those who have previously participated in pre- and post-test assessment. These students, now in their M4 year, were given the same 18-question assessment used in the pre- and post- test. In order to control for improvement in diabetes that might occur during sub-interships training or other clinical rotations, the long-term assessment was timed such that it was administered after the majority of students would have completed their subinternship training (similar to the control group). Students completing the long-term assessment were entered into a raffle for $25 Amazon.com gift cards to encourage participation.

### Control group

The control group consisted of M4 students at UMMS during the 2012–2013 academic year (2012–2013 cohort). These students, recruited via group email during the 2^nd^ half of their 4^th^ year of medical school (2012–2013 school year), did not have hospital diabetes management as a formal component of their curriculum. They completed the same 18-question assessment tool as the experimental group and were also incentivized for participation with a $25 Amazon.com gift card raffle.

### Statistical analysis

For the knowledge-based questions, the score was calculated by summing the number of correct responses out of a maximum possible score of 15. For the confidence questions, responses were scored using a range from 1–4, 1: extremely unconfident and 4: extremely confident. The short-term (pre- and post-test) responses for the experimental group were compared using a two-tailed paired *t*-test. To compare the experiemental group to the control group, a student’s *T*-test and Wilcoxon Mann Whitney test were used. Comparison of responses on individual questionnaire items were made using a Fischer’s exact test.

Finally, to determine the relationship between the knowledge score and confidence score a proportional odds model was fitted to the data. The model included the main effects group and aggregate knowledge score and the interaction terms. All statistical analysis was performed using the statistical software SPSS version 19 or the Real Statistics Resource Pack for Excel.

## Results

With regards to the short-term knowledge assessment, 69 students participated in the pre- and post-test assessment using the audience reponse system. Of these, the 53 students who completed at least half of both the pre- and post-test questions were included the in the analysis; on average students included in the study attemped 13.2 +/- 1.8 pre-test questions and 14.2 +/- 1.4 post-test questions. The mean score out of 15 questions was 7.7 +/- 2.7 (51.2%) on the pre-test and 11.4 +/- 1.8 (75.7%) on the post-test (*p* < 0.01). If missing responses are excluded this difference remains significant with an average score of 58% for the pre-test and 80% for the post-test (*p* < 0.01). See Table 1 for the percentage correct for individual item among the 15 multiple-choice knowledge questions.

**Table 1:** Percentage of correct responses to individual questions according to group

	Experimental Group			Control Group	*P*-value
	Pre-Test	Post-Test	6 months-Post	M4 Controls	6 months-Post vs Control
Which answer choice contains *only* basal insulin?	60	92	85	84	0.84
Which answer choice contains *only* bolus insulin?	66	85	84	83	1
What is the difference between prandial insulin and correction insulin?	68	89	92	94	0.76
What is the difference between basal insulin and bolus insulin?	83	87	87	88	0.82
When a patient is made NPO, which type of insulin order should *always* be held?	58	91	80	80	1
For a patient with Type 1 Diabetes, which type of insulin order should *never* be completely held?	66	98	88	81	0.29
Upon admitting a patient with Type 2 Diabetes to a general care unit, what is the appropriate initial strategy for oral anti-diabetes medications?	49	94	72	51	***0.01***
What is the approximate duration of action of regular insulin?	25	30	24	36	0.1
What is the approximate duration of action of insulin aspart/Novo log and lispro/Humalog?	13	23	28	24	0.6
What is the approximate duration of action of insulin glargine/Lantus?	55	89	65	65	1
What is the approximate duration of action of NPH insulin?	51	42	40	32	0.34
As a starting point, which range of calculations can you use to estimate insulin total daily dose (TDD) for a patient with diabetes?	47	92	52	39	0.09
As a starting point, how should total daily dose (TDD) of insulin be divided?	60	83	59	59	1
What is 70/30 insulin?	51	60	47	56	0.23
Systemic steroids impact all blood sugars the *greatest* impact is on which?	15	81	12	4	0.08
Overall	51	76	61	58	0.3

For the long-term knowledge durability assessment, surveys were collected from 100 students from the control group (2012–2013 cohort) and 75 students in the experimental group (2013–2014 cohort). This represented a 60% response rate for the control group and a 44% response rate from the experimental group. Given that the audience response system is annonymous we cannot determine the degree of overlap between the the students who completed the immediate pre- and post- test assessment and those who participated in the assessment 6-months after the seminar. However, both are representative samples out of the estimated 175 students in total who attended the seminar. The mean number of correct answers (out of 15 questions) in the control group was 8.8 ± 2.5 (58%) compared to 9.2 ± 2.3 (61%) in the experimental group at 6-months after the seminar. Percentage correct for individual items are listed in Table 1. The experimental group in the immediate post-test scored significantly higher than the control group with *p* < 0.01. However, there was no significant difference between the experimental group at 6-months and the control group. (*p* = 0.30).

There was also no significant difference in student-reported confidence in managing type 2 diabetes between the experimental group at 6-months after the seminar and the control group. Confidence scores for managing type 2 diabetes trailed scores for managing blood pressure and electrolyte imbalances in the hospital (Table 2). Confidence level in treating type 2 diabetes in the hospital increased with increasing knowledge score for the 15 diabetes knowledge questions (OR = 1.84, *P* = 0.02) across cohorts. There was no similar increase in the confidence level in treating electrolyte imbalance and blood pressure.

**Table 2:** Mean student-reported confidence scores

	Control group (*n* = 100)	Experimental group (*n* = 75)	*P*-value
Confidence level			
Managing Type 2 Diabetes	2.01 ± 0.77	2.17 ± 0.76	0.16
Managing electrolyte imbalances	2.48 ± 0.70	2.56 ± 0.70	0.57
Managing blood pressure	2.73 ± 0.55	2.80 ± 0.68	0.45

Items assessed on a scale of 1–4, where 1 = extremely UNconfident/always need supervisor assistance, 2 = somewhat UNconfident/often need supervisor assistance, 3 = somewhat confident/occasionally need supervisor assistance, 4 = extremely confident/almost never need supervisor assistance

*P*–value was obtained by Wilcoxon Mann Whitney test

## Discussion

The addition of an interactive seminar as a madantory part of the 3^rd^ year medical school curriculum resulted in short-term improvement in knowledge of hospital diabetes management, as evidenced by a significant improvement in scores on multiple choice questions from the pre-test to the post-test assessment. Students on the post-test also scored significantly higher than the control group of students from the preceding medical school class who did not participate in the seminar. However, when students were given the same set of questions 6 months later, scores declined, and were no better than the control group. Several previous studies assessing short-term educational interventions and exclusively evaluating short-term outcomes have reported similar findings [15, 21, 27, 28]. This may represent effective presentations and/or the advantages of short-term memory.

Very few studies have evaluated the long-term impact of an intervention on trainee knowledge of hospital diabetes management. Our study suggests that evaluating only short-term post-intervention data can overestimate the impact of a single-session of educational intervention. We were asked to bolster the medical student curriculum by specifically focusing on hospital diabetes management. The authors (TWB, JJI, RYG) had noticed a lack of knowledge and comfort managing diabetes in the hospital amongst trainees in our institution, so we chose to assess not only the short-term gains in knowledge and confidence, but also the long-term gains. Despite an immediate improvement, students scored no better when reassesed 6 months later in comparison to the control cohort (which did not have the inpatient diabetes seminar in their curriculum).

Our study fills an important gap in the existing literature by demonstrating a lack of durability of improvement following an interactive seminar. This may explain the disconnect between the positive findings in many of the educational intervention studies and the fact that many trainees still report feeling ill-equipped to manage the diabetes scenarios they encounter in the hospital. Since the hospital teams are not comfortable managing diabetes there is not much opportunity to address this gap during medical student clerkships on the inpatient floors.

Our study results also that medical educational interventions in hospital diabetes should should consider a more longitudinal addition to the curriculum, perhaps with an even greater component of active learning, an idea reflected in the findings of a qualitative study of medical student learning by Luscombe and Montgomery [29]. We point out that diabetes and hyperglycemia is ubiquitous in the hospital environment, and despite ongoing exposure to the topic, knowledge gains after our interactive seminar were not durable. One could hypothesize that topics encountered less frequently may suffer more from loss of knowledge gains. It may not be reassuring, but rather alarming, to learn that putting substantial effort into bolstering a curriculum with a single seminar may not “move the needle” much in the long run.

To our knowledge there has only been one previously published study which examined the durability of a short-term educational intervention to improve inpatient diabetes knowledge. Tamler and colleagues used computer-based modules to educate internal medicine residents on hospital diabetes management. While they did find their intervention durably improved scores on a multiple choice question assessment, they were unique in that they administered a refresher course to residents several months after completing the initial course. They noted that topics that were not included in the refresher and not frequently encountered on the wards had declining scores over time. Their study supports our data and suggests that the addition of a refresher course could be one way to improve the durability of our initial knowledge gains [30].

The longest follow up after an intervention associated with hospital diabetes management education was a study using a two-pronged approach. One was an endocrinologist rounding with general medicine residents two times a day for 2 weeks on the diabetes patients admitted to the hospital. The second was to provide medicine residents with pocket cards outlining hospital diabetes management guidelines. Their dual effort improved diabetes knowledge and also reduced hyperglycemia in hospitalized diabetes patients over a 12 month period. This approach delivered continuous inpatient diabetes education over a sustained period of time and therefore had the ability to cover and reinforce various glucose management scenerios [31].

Our study indeed has limitations. It is a single-center study evaluating a single educational intervention and targets third year medical students. Results may not be broadly generalizable to other institutions, types of short-term interventions, or levels of trainees. For example, it may be that our efforts to target the diabetes knowledge and confidence gap across disciplines was targeted too early in their training to fully engage our learners, who did not yet have broad clinical experience with hospital diabetes management. An additional limitation is that response rates were low but still are acceptable rates for survey literature. We also did not have the authority to mandate 100% audience participation during the seminar with using the audience response system, nor the authority to mandate completion of the survey 6 months later.

Future studies are needed to address more broadly (at multiple institutions, various methods of instruction and learning, different groups of trainees) educational interventions that reinforce knowledge and lead to *durable* gains. Additionally, we suggest that this problem is not limited to the topic of hospital diabetes management, but is more pervasive in medical education. It may also be useful to randomize groups of learners to variable interventions differing in scope, target audience and longitudinal nature.

## Conclusions

Adding a single seminar on hospital diabetes management in the M3 year boosted immediate post-seminar performance compared to pre-seminar knowledge. This intervention did not durably improve medical student knowledge or change confidence levels at a 6-month evaluation. Repetative interactions with greater focus on the topic during clinical rotation and other sustained methods of exposure need to be evaluated. Furthermore, knowledge and confidence managing diabetes in the hospital go hand in hand, suggesting the possibility that interventions that increase one may help the other.

## Additional file

Additional file 1: Hospital Diabetes Management Questions. (DOCX 16 kb)

## Abbreviations

M3 year: 3^rd^ year of medical school
M4 year: 4^th^ year of medical school
UMMS: University of Michigan Medical School
