# Supplementary material for: Can a single interactive seminar durably improve knowledge and confidence of hospital diabetes management?
Source: Clin Diabetes Endocrinol. 2016 Dec 1;2:20. doi: 10.1186/s40842-016-0038-4 (PMC5471697; doi:10.1186/s40842-016-0038-4)
Supplement: Additional file 1: — Hospital Diabetes Management Questions. (DOCX 16 kb) [file 40842_2016_38_MOESM1_ESM.docx]

**Additional file 1**

**Hospital Diabetes Management Questions**

1. Which answer choice contains ONLY *basal* insulins?
   1. Regular, lispro/Humalog, aspart/Novolog
   2. Regular, NPH, aspart/Novolog
   3. NPH, glargine/Lantus, detemir/Levemir
   4. Glargine/Lantus, regular, lispro/Humalog
2. Which answer choice contains ONLY *bolus* insulins?
   1. Regular, lispro/Humalog, aspart/Novolog
   2. Regular, NPH, aspart/Novolog
   3. NPH, glargine/Lantus, detemir/Levemir
   4. Glargine/Lantus, regular, lispro/Humalog
3. What is the difference between *prandial* insulin and *correction* insulin?
   1. Prandial = meal coverage, correction = treat high blood sugars
   2. Prandial = fasting insulin needs, correction = treat high blood sugars
   3. Prandial = treat high blood sugars, correction = meal coverage
   4. Prandial = meal coverage, correction = fasting insulin needs
4. What is the difference between *basal* insulin and *bolus* insulin?
   1. Basal = fasting insulin needs, bolus = correction insulin only
   2. Basal = meal insulin only, bolus = correction insulin only
   3. Basal = meal and correction insulin, bolus = fasting insulin needs
   4. Basal = fasting insulin needs, bolus = meal and correction insulin
5. When a patient is made NPO, which type of insulin order should ALWAYS be *held/stopped/discontinued*?
   1. Basal insulin
   2. Prandial insulin
   3. Correction insulin
   4. Sliding scale insulin
6. For a TYPE 1 diabetic patient, which type of insulin order should NEVER be completely *held/stopped/discontinued*?
   1. Basal insulin
   2. Prandial insulin
   3. Correction insulin
   4. Sliding scale insulin
7. Upon admitting a TYPE 2 diabetic patient to a general care unit, what is the appropriate initial strategy for oral anti-diabetic medications?
   1. Metformin should be *held/discontinued*
   2. Sulfonylureas such as glipizide/Glucotrol should be *held/discontinued*
   3. Thiazolidinediones such as pioglitazone/Actos should be *held/discontinued*
   4. All should be *held/discontinued*
8. What is the approximate duration of action of regular insulin?
   1. 1-2 hours
   2. 3-4 hours
   3. 5-6 hours
   4. 7-8 hours
9. What is the approximate duration of action of insulins aspart/Novolog, lispro/Humalog, and glulisine/Apidra?
   1. 1-2 hours
   2. 3-4 hours
   3. 5-6 hours
   4. 7-8 hours
10. What is the approximate duration of action of insulin glargine/Lantus?
    1. 6-8 hours
    2. 9-12 hours
    3. 15-18 hours
    4. 20-24 hours
11. What is the approximate duration of action of NPH insulin?
    1. 6-8 hours
    2. 9-12 hours
    3. 15-18 hours
    4. 20-24 hours
12. As a starting point, which range of calculations can you use to estimate total daily dose (TDD) of insulin for a diabetic patient?
    1. 0.03 units/kg/day to 0.1 units/kg/day
    2. 0.1 units/kg/day to 0.3 units/kg/day
    3. 0.3 units/kg/day to 1.0 units/kg/day
    4. 1 units/kg/day to 3 units/kg/day
13. As a starting point, how should total daily dose (TDD) of insulin be divided?
    1. 33% basal, 67% bolus
    2. 50% basal, 50%bolus
    3. 67% basal, 33% bolus
    4. 75% basal, 25% bolus
14. What is 70/30 insulin?
    1. A mixture of 70% glargine/Lantus insulin and 30% fast-acting insulin
    2. A mixture of 70% fast-acting insulin and 30% glargine/Lantus
    3. A mixture of 70% NPH insulin and 30% fast-acting insulin
    4. A mixture of 70% fast-acting insulin and 30% NPH
15. Systemic steroids impact all blood sugars. The GREATEST impact is on which?
    1. Overnight glucoses
    2. Fasting morning glucoses
    3. Pre-meal glucoses
    4. Post-meal glucoses
16. How *confident* are you with managing TYPE 2 DIABETES in the INPATIENT setting?
    1. Extremely UNconfident = I *always* need help from a supervising physician
    2. Somewhat UNconfident = I *often* need help from a supervising physician
    3. Somewhat Confident = I *occasionally* need help from a supervising physician
    4. Extremely Confident = I *almost never* need help from a supervising physician
17. How *confident* are you with managing ELECTROLYTE IMBALANCES in the INPATIENT setting?
    1. Extremely UNconfident = I *always* need help from a supervising physician
    2. Somewhat UNconfident = I *often* need help from a supervising physician
    3. Somewhat Confident = I *occasionally* need help from a supervising physician
    4. Extremely Confident = I *almost never* need help from a supervising physician
18. How *confident* are you with managing BLOOD PRESSURE in the INPATIENT setting?
    1. Extremely UNconfident = I *always* need help from a supervising physician
    2. Somewhat UNconfident = I *often* need help from a supervising physician
    3. Somewhat Confident = I *occasionally* need help from a supervising physician
    4. Extremely Confident = I *almost never* need help from a supervising physician
